# Spatial-Numerical Associations Enhance the Short-Term Memorization of Digit Locations

**DOI:** 10.3389/fpsyg.2018.00636

**Published:** 2018-05-07

**Authors:** Catherine Thevenot, Jasinta Dewi, Pamela B. Lavenex, Jeanne Bagnoud

**Affiliations:** Institute of Psychology, University of Lausanne, Lausanne, Switzerland

**Keywords:** numerical cognition, space, mental number line, SNARC, short-term memory, long-term memory

## Abstract

Little is known about how spatial-numerical associations (SNAs) affect the way individuals process their environment, especially in terms of learning and memory. In this study, we investigated the potential effects of SNAs in a digit memory task in order to determine whether spatially organized mental representations of numbers can influence the short-term encoding of digits positioned on an external display. To this aim, we designed a memory game in which participants had to match pairs of identical digits in a 9 × 2 matrix of cards. The nine cards of the first row had to be turned face up and then face down, one by one, to reveal a digit from 1 to 9. When a card was turned face up in the second row, the position of the matching digit in the first row had to be recalled. Our results showed that performance was better when small numbers were placed on the left side of the row and large numbers on the right side (i.e., congruent) as compared to the inverse (i.e., incongruent) or a random configuration. Our findings suggests that SNAs can enhance the memorization of digit positions and therefore that spatial mental representations of numbers can play an important role on the way humans process and encode the information around them. To our knowledge, this study is the first that reaches this conclusion in a context where digits did not have to be processed as numerical values.

## Introduction

Spatial-numerical associations (SNAs) have been extensively studied since Dehaene et al.’s (1993) discovery that for individuals from Western cultures, decisions on small numbers are taken quicker with the left than the right hand, and quicker on large numbers with the right than the left hand. This SNA of response codes, or SNARC effect, was originally interpreted as the result of spatial congruency between the response hand and the position of numbers on a left-to-right mental number line (MNL) stored in long-term memory and representing increasing magnitudes of numbers (Moyer and Landauer, 1967).

The orientation of the MNL could be derived from cultural factors and especially from the direction of reading. This interpretation is supported by several studies showing that SNARC effects observed in Western participants can be reduced or even inversed in right-to-left readers (Dehaene et al., 1993; Zebian, 2005; Shaki et al., 2009). However, it has been shown that western children already preferentially represent numbers from left to right rather than right to left before school entry (Opfer et al., 2010; Thevenot et al., 2018).

For some authors, this kind of oriented representations of numbers in preliterate children stem nonetheless from environmental reading conventions (Shaki et al., 2012; Göbel et al., 2018) whereas, for others, the direction in which pre-schoolers count objects is not necessarily linked to their knowledge about cultural reading practice (Patro and Haman, 2017). In fact, some researchers even adopt a nativist view and argue that babies and animals share the intuition that small quantities are represented on the left of a mental-spatial continuum. For example, it has been shown that 7-month-old children prefer left-to-right arrangements of non-symbolic numerosities (sets of dots) in ascending rather than descending order (de Hevia et al., 2014) or that chicks prefer a panel placed on their left when it represents a smaller numerosity than a target, and a panel placed on their right when it represents a larger numerosity than the target (Rugani et al., 2015).

In human adults, the existence of such SNAs has been revealed by numerous studies using different paradigms (Fischer, 2001; Shaki et al., 2012; Masson et al., 2013; Masson and Pesenti, 2014; Mathieu et al., 2016, 2017; see Fischer and Shaki, 2014 for a review). For example, Fischer et al. (2003) showed that a target presented on the left side of a computer screen is detected faster when it is preceded by small rather than large numbers and, conversely, that a target on the right side of the screen is detected faster when it is preceded by larger numbers. Small and large numbers seem therefore to draw the attention of individuals to their left and right visual fields, respectively. These results have been replicated several times, notably by Bonato et al. (2009) or Dodd et al. (2008). However, Galfano et al. (2006) suggest that these attentional biases do not occur automatically but only when participants have to explicitly process the digits for numerical purposes.

In sum, even if the innate nature of the mental number line or its automaticity remains under debate, the fact that numbers and space are associated is now well established. However, as noted by McCrink and Shaki (2016), less is known about how SNAs affect the way individuals process their environment, especially in terms of learning and memory. To address this question, we have investigated the potential effects of SNAs in a memory task. Specifically, we aimed to determine whether the spatial position of digits is better memorized when they are congruent rather than incongruent with the positions of numbers on the internalized MNL.

This question was recently addressed by Gut and Staniszewski (2016) who presented digits to the right or to the left of a central fixation point. When the digits disappeared, the fixation point was replaced by one of the two digits, and participants had to recall the location where the target digit had been previously presented. The authors showed that memory performance (i.e., shorter RTs and lower error rates) was better for “small” as compared to “large” digits when they were positioned to the left of the fixation point. However, the reverse congruency effect was not observed. In other words, when the digits were presented to the right of the fixation point, both “small” and “large” digits led to similar memory performance. The authors offered several explanations for these results. First, they suggested that a congruency effect only for small numbers constitutes evidence that the magnitude of numbers plays a role in SNARC-like effects. According to the authors, small numbers such as one or two might be encountered more often and might better catch individuals’ attention than larger numbers (Cai and Li, 2015) and this would partly explain why congruency effects were only obtained for very small one-digit numbers. Nevertheless, the authors note that, in opposition to these assumptions, smaller numbers might not be encountered more often than larger numbers (e.g., Dehaene and Mehler, 1992) but that individuals might react faster to higher than lower magnitude numbers (e.g., Krause et al., 2017). Moreover, Gut and Staniszewski (2016) observed that, independent of number magnitudes, their participants were faster to recall the location of digits when they were displayed to the right rather than to the left of the fixation point. However, because digits to the right of the fixation point were always responded to with the right hand (and digits to the left of the fixation point responded to with the left hand) and given the fact that all their participants were right-handed, better dexterity with the right hand might explain their results. All in all, and despite the interesting question raised by the authors, a coherent explanation of their findings was not obvious. Their conclusion that “the spatial representation of numbers on the MNL are crucial for retrieval of numbers presented on the left and that the responses to the numbers presented on the right are generally faster and more correct irrespective of their congruency” (p. 203) is difficult to reconcile with the results of the numerous studies showing faster processing for large numbers when they are presented in the right visual field of participants, including the famous original SNARC effect.

The question of potential effects of digit processing in a memory task has also been addressed by McCrink and Galamba (2015) in a series of experiments in which spatial locations had to be memorized. Participants were presented with a series of sequentially highlighted spatial locations on a grid and their task was to repeat the sequence by touching the locations on a computer screen. The locations could appear from left to right, from right to left or randomly in the grid. In one of the conditions, symbolic numerals were associated with the locations but, contrary to the authors’ expectations, there was no advantage of the left-to-right over the right-to-left flow for the recall of the locations. However, it is possible that in McCrink and Galamba’s (2015) task, the sequential movement of the digits in a two-dimensional space might preclude them from being influenced by the MNL where number are represented strictly one-dimensionally.

In sum, the question of whether there are improvements in the encoding and recall of numbers when their spatial positions are congruent with MNL orientation remains unanswered. The object of the current study is to further investigate this question with a memory game in which adult participants had to match pairs of identical numbers. The game was presented on a computer screen where two rows of cards hiding numbers were displayed. The first row of nine cards was created by using the nine digits from 1 to 9. In the congruent condition, the digits 1–4 were randomly placed to the left of the five, which was placed in the exact middle of the row, and the digits 6–9 were randomly placed to its right. In the incongruent condition, small digits from 1 to 4 were placed to the right of the five and larger digits from 6 to 9 were placed to its left. Finally, in the random condition, digits were randomly placed on the first row. For all the configurations, the digits from 1 to 9 were pseudo-randomly positioned on the second row. If the organization of numbers on the MNL can enhance the memorization of numerical material, individuals should perform better in the congruent than in the incongruent and random conditions. Moreover, and conversely, if the organization of numbers on the MNL can interfere with the memorization of numerical material, individuals should also perform better in the random than in the incongruent condition in which the presentation of numbers conflicts maximally with the representations of numbers on the MNL.

## Materials and Methods

### Participants

Twenty-five right-handed undergraduate students in Psychology at the University of Lausanne participated for course credit. Participants were aged between 18 and 32 years (mean: 21.64 years) and five of them were men.

### Material and Procedure

The task we designed was an adaptation of the classic memory game where cards are placed face down in front of a player who has to find matching pairs. To do so, he or she has to turn the cards face up and encode the symbol that occurs on the card and its position before turning them face down again. In our adaptation, participants were presented with two rows of nine squares, representing the cards, on a computer screen. The symbols that had to be encoded were digits from 1 to 9. Participants were first introduced to the task and instructed how to play with a shorter version of the task using geometrical shapes instead of digits.

During each game, participants were presented with two rows of cards that they could turn face up and down by clicking on them. The participants had been instructed that the first card to turn face up was the card at the leftmost position of the first row. Once the digit was seen, the card had to be turned face down again and the card immediately to its right had to be turned face up. This rule had to be applied until the end of the game and, at the end of the first row, it was the card at the leftmost position of the second row that had to be turned over. However, if the participant thought that she had already seen the digit on the card, she could try to find it by returning to and overturning a previous card. If she succeeded, the two cards stayed visible on the screen and she could continue with the game. If she made a mistake, she had to turn the last card (the incorrect choice) face down and then could either attempt to find the matching card again, or she could give up for this pair and continue the game by turning over the next card in the row. Participants were not aware that it was not possible to find a pair before the end of the first row. If all the pairs were not found when the last card was turned up, the participant was free to return any card of the game. A perfect game, without any mistakes, could be completed in 27 moves, corresponding to nine moves on the first line to discover the positions of the nine digits and 18 moves to match the pairs (i.e., one move to overturn each card on the second row and one move to match it to a card in the first row).

**FIGURE 1:**
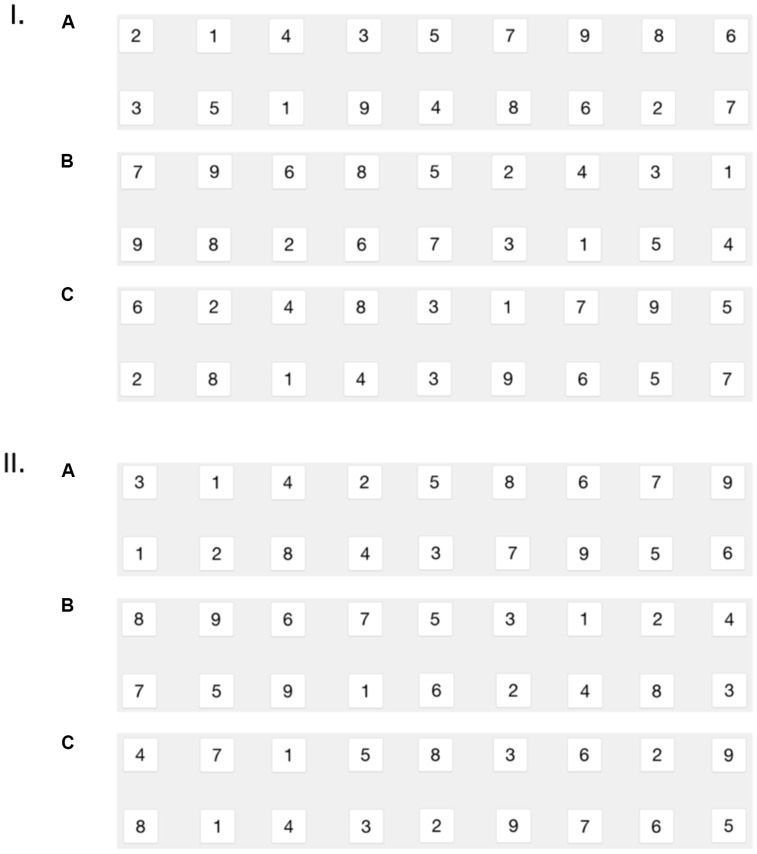
The two versions of the randomized material in the memory game (I and II) with the three different configurations: congruent **(A)**, incongruent **(B)**, and random **(C)**.

As described in the Introduction, the first row of cards could be in a congruent, incongruent or random arrangement. In the congruent condition, the digits 1–4 were randomly placed to the left of the five, which was placed in the middle of the row, and the digits 6–9 were randomly placed to its right. In the incongruent condition, the digits of the congruent condition were replaced by digits using the inverse ordering of numbers (i.e., 1 replaced by 9, 2 replaced by 8, 3 replaced by 7, 4 replaced by 6 and vice versa). Finally, in the random condition, digits were all placed pseudo-randomly in the first row with the rule that all small or all large digits could not be positioned on the same side of the row. For all the conditions, the digits from 1 to 9 were pseudo-randomly positioned on the second row with the rule that two matching symbols were separated at least by five sequential positions (e.g., the number six could not be the last digit on row one and the first digit on row two). In order to minimize the risk of accidental biases, two versions of the randomized material were created for each condition and all participants played the same randomized versions of the game in all three conditions (**Figure 1**). Therefore, participants played the game six times (3 conditions × 2 versions). For each of the versions, the three conditions were presented in a counterbalanced manner to participants, so that 1/3 played the game with the congruent condition first, 1/3 played the game with the incongruent condition first, and 1/3 played the game with the random condition first. For each participant, we measured the time required to complete each of the six games and the number of moves realized to do so.

## Results

### Solution Times and Accuracy

A repeated measures ANOVA with configuration (congruent, incongruent, and random) as a within-subjects factor revealed differences in the solution times, *F*(2,48) = 4.81, $\eta_{p}^{2}$ = 0.17, *p* = 0.01, and the number of moves, *F*(2,48) = 4.44, $\eta_{p}^{2}$ = 0.16, *p* = 0.02 (**Table 1**).

**Table 1:** Solution times (in seconds) and number of moves (and standard-deviations) for the congruent, incongruent, and random configurations in the memory game.

	Solution times	Number of moves
Congruent	55.71 (14.74)	31.86 (3.59)
Incongruent	64.95 (24.43)	34.64 (7.24)
Random	65.41 (18.49)	35.62 (5.39)

Because the SNA hypothesis allowed us to put forward precise predictions, one-sided planned comparisons with Bonferroni corrections were conducted to compare solution times between the congruent condition and the other two conditions. They revealed that solution times were shorter in the congruent (55.71 s) than in the incongruent (64.95 s), *z* = 2.62, *p* = 0.01, and random conditions (65.41 s), *z* = 2.75, *p* < 0.01. For the comparison between the incongruent and random conditions, a one-sided planned comparison with Bonferroni correction showed no significant difference between the two conditions, *z* = 0.13, *p* = 1. An additional Bayesian analysis on this difference revealed substantial evidence for this absence of effect (*BF*_10_ = 0.28).

The same pattern of results was obtained for the number of moves. Indeed, participants completed the game in a fewer number of moves in the congruent (31.86 moves) than in the incongruent (34.64 moves), *z* = 2.12, *p* = 0.05, and random conditions (35.62 moves), *z* = 2.87, *p* < 0.01. Again, there was no significant difference in the number of moves between the incongruent and random conditions, *z* = 0.75, *p* = 0.68 and the Bayesian analysis on this difference revealed substantial evidence for this absence of effect (*BF*_10_ = 0.325).

## Discussion

In this research we were interested in the question of whether representations of numbers on a mental number line can influence the short-term memorization of number positions displayed in front of a participant. To this aim, we adapted the classic memory game and showed that the positions of digits representing smaller and larger numbers were more easily recalled when they were presented on the left and on the right of the display, respectively, than when it was the reverse or when the digits were randomly positioned. This finding suggests that the spatial mental representation of numbers in a left-to-right orientation can facilitate the memorization of digit localizations. An oriented representation of numbers activated during the memory task could have indeed constituted a framework that helped individuals to encode and recall the positions of the digits. Importantly, in the task that we designed the digits to be memorized did not need to be processed as numbers. It seems therefore that SNAs can be activated in absence of explicit processing of number magnitudes. Interestingly, these congruency effects were observed despite possible interferences due to “micro-incongruences” on both sides of our display. In fact, due to the constraints of the task, the digits could not be presented strictly in the canonical order and therefore, within one side, a smaller digit could be preceded or followed by a larger digit. Obviously these “micro-incongruences,” which were present in the three conditions of our experiment, did not significantly impact our results.

Contrary to our expectations, we did not observe differences in memory performance between the random and the incongruent conditions. Thus, it appears that a presentation of numbers that maximally conflict with the organization of the MNL is not more detrimental to memory performance than a less incongruent (e.g., random) presentation of numbers. This result may lead to a number of different interpretations. First, it is possible that the MNL is not automatically activated as soon as numerals are presented to the cognitive system and that it is activated only when the presented configurations match an ordered sequence of numbers stored in long-term memory. In other words, the MNL would not be activated when numbers are encountered in an order that does not match any long-term memory representations, hence the lack of difference between the incongruent and the random conditions. According to this interpretation, it would not even be necessary to assume that numbers are organized from left-to-right in long-term memory but simply that numbers are ordered in long-term memory. Indeed, according to an alternative account of SNARC effects, associations between space and numbers are due to the characteristics of maintenance of information in short-term memory rather than magnitude representations in long-term memory (e.g., van Dijck and Fias, 2011; van Dijck et al., 2014; Abrahamse et al., 2016). A strong empirical argument for this view is that any ordered information maintained in short-term memory, such as months of the year, letters of the alphabet or even a list of words just memorized, is represented from left to right and is subject to SNARC effects (e.g., Gevers et al., 2003, 2004). Within this framework, the organization of numbers in the congruent condition of our experiment would convoke a non-oriented ordered sequence of numbers from long-term memory and the ordered sequence would temporarily be oriented from left to right in short-term memory. This transitory representation of numbers would serve as a framework to encode and recall the position of digits. In this case, our results would contradict Dehaene’s seminal interpretation of SNARC effects (Dehaene et al., 1993) according to which the magnitude of number is automatically activated and inherently associated with space.

An alternative interpretation of the absence of differences in memory performance between the incongruent and the random conditions could be that in both cases the MNL is automatically activated but that the difficulty to memorize random configurations equates with the difficulty to inhibit information in total conflict with the MNL organization. Further experiments designed to directly contrast these alternative interpretations will have to be conducted in the future. One possibility would be to examine potential MNL priming effects across conditions. Indeed, when the incongruent and random conditions are presented before the congruent condition, and if the MNL is automatically activated in these conditions, priming effects of the MNL should be observed in the subsequent congruent condition. The improvement in memory performance that we observed in the congruent condition of our experiment should therefore be increased. Conversely, even if the MNL is not automatically activated in the incongruent and random conditions, it is likely to be activated after participants performed the task in the congruent condition. In this case, we should observe priming effects of the MNL on the incongruent and random conditions when they are performed after the congruent condition but no priming effects of the MNL on the congruent condition when it is performed before the random and incongruent conditions. Unfortunately, such analyses are impossible with the present data set because participants played too few games in each condition.

Finally, a last alternative interpretation of our results is that enhanced memory performance in the congruent condition is not due to any ordering of numerical information in short-term memory but only to the number themselves, which could trigger the attention of participants on the left or of the right attentional fields depending on their size (i.e., on the left for small numbers and on the right for larger ones) (Gevers et al., 2006; Proctor and Cho, 2006; Santens and Gevers, 2008). Nevertheless, we think that the lack of difference in memory performance between the incongruent and random conditions argues against this interpretation. Indeed, the conflicts between attentional biases triggered by the magnitude of numbers and the position of the numbers in the memory game are maximal in the incongruent condition and memory performance should therefore be worst in this condition than in the random condition. Still, this line of reasoning is based on a lack of effect and has to be considered with care.

## Conclusion

We have shown that SNAs can enhance the memorization of digit positions and thus that spatial mental representations of numbers could play an important role in the way that humans process and encode the information around them. To our knowledge, this study is the first that reaches this conclusion in a context of a memory task where the digits did not have to be processed as numerical values. This suggests that either Arabic numerals cannot be perceived as pure symbols lacking numerical characteristics, or that individuals can consciously evoke numerical knowledge associated with digits when it is potentially helpful for them.

## Ethics Statement

Signed informed consent forms were obtained from all our participants before they entered the study. The experiment reported in the present manuscript has been conducted in compliance with the Swiss Law on Research involving human beings and because only behavioral data were collected in a non-vulnerable population of adults, the approval of the Canton de Vaud ethic committee was not required. This study was carried out in accordance with the recommendations of the Ethics Committee of the University of Lausanne with written informed consent from all subjects in accordance with the Declaration of Helsinki.

## Author Contributions

CT had the original idea of the research. CT and JB wrote a first draft of the manuscript and PL edited the manuscript. JB conducted the experiment. All the authors contributed to the conception of the experiment.

## Conflict of Interest Statement

The authors declare that the research was conducted in the absence of any commercial or financial relationships that could be construed as a potential conflict of interest.
